# Comparison of Two Apnea Test Methods, Oxygen Insufflation and Continuous Positive Airway Pressure During Diagnosis of Brain Death: Final Report

**DOI:** 10.1007/s12028-018-0608-7

**Published:** 2018-09-12

**Authors:** Joanna Solek-Pastuszka, Jowita Biernawska, Waldemar Iwańczuk, Klaudyna Kojder, Kornel Chelstowski, Romuald Bohatyrewicz, Marcin Sawicki

**Affiliations:** 1 Department of Anaesthesiology and Intensive Care, Pomeranian Medical University in Szczecin, Unii Lubelskiej 1, 71-252 Szczecin, Poland; 2 Department of Anaesthesiology and Intensive Therapy, Regional Hospital in Kalisz, 62-800 Kalisz, Poland; 3 Department of Laboratory Diagnostics, Pomeranian Medical University in Szczecin, Al. Powstańców Wlkp. 72j, 70-111 Szczecin, Poland; 4 Department of Diagnostic Imaging and Interventional Radiology, Pomeranian Medical University in Szczecin, Unii Lubelskiej 1, 71-252 Szczecin, Poland

**Keywords:** Apnea test, Brain death, Brain death diagnosis, Continuous positive airway pressure

## Abstract

**Introduction:**

Deterioration of the pulmonary function after the apnea test (AT) conducted with the classic oxygen insufflation AT (I-AT) is often observed during the brain death (BD) diagnosis procedure. In the present study, two AT methods were compared before a method is recommended for the currently revised Polish BD criteria.

**Methods:**

Classic I-AT and continuous positive airway pressure AT (CPAP-AT) were performed in 60 intensive care unit patients. I-AT was performed at the end of two series of clinical tests, and approximately 1–1.5 h later, after BD was confirmed, a different method, CPAP-AT with 100% FiO_2_ and CPAP value of 10 cm H_2_O provided by a ventilator in CPAP mode was performed. The patients in I-AT and CPAP-AT groups were further divided into two subgroups: non-hypoxemic (NH) with good lung function before AT (PaO_2_/FiO_2_ index ≥ 200 mmHg) and hypoxemic (H) with poor lung function (PaO_2_/FiO_2_ index < 200 mmHg). PaO_2_ and PaCO_2_ were recorded prior to I-AT and CPAP-AT at time-point one (T1), 5 min after each test at time-point two (T2), and after 10 min prior to the end of tests at time-point three (T3). The I-AT NH subgroup consisted of 50 patients, and CPAP-AT NH subgroup 43 patients. The I-AT H subgroup consisted of 10 patients, and the CPAP-AT H subgroup 17 patients.

**Results:**

In the I-AT NH subgroup, a gradual decrease in PaO_2_/FiO_2_ was observed throughout the AT but not in the CPAP-AT NH subgroup. The PaO_2_/FiO_2_ ratio during the AT in the CPAP-AT H group was stable with a slight tendency to increase but not in the I-AT H group. During the first 5 min of the AT, the mean increase in CO_2_ was approximately 5 mmHg/min. Most patients in all groups met the AT criteria after 5 min of the test.

**Conclusions:**

The results from the study show that I-AT may compromise pulmonary function in some cases and is one of the reasons for the recommendation of a safer option, CPAP-AT, in the currently revised Polish BD criteria. During AT, the mean CO_2_ increase rate was 5 mmHg/min, which, in most patients, would allow the test to be completed after just 5 min.

## Background

Polish brain death (BD) criteria are currently under review. They are generally similar to US and other European guidelines based on the recognition of irreversible catastrophic brain damage and two series of clinical examinations confirming brainstem areflexia and apnea [1, 2]. In Poland, instrumental ancillary tests are needed when confounders are present and in children under 2 years of age. All national BD diagnosis guidelines are periodically reviewed, but despite many efforts, they have not been unified to date [1].

Current debates between medical practitioners in Poland cover many aspects, including the evolution of BD concepts [3] and the implementation of new diagnostic techniques such as computed tomography (CT) angiography and perfusion [4]. Practical aspects of clinical examinations including the apnea test (AT) in patients with poor lung function or extracorporeal membrane oxygenation support are extensively discussed [5].

The AT is one of the most important examinations during BD diagnosis and usually performed at the end of each of two series of clinical examinations. The most common, classical insufflation AT (I-AT) is currently recommended in Polish BD criteria published in 2007 [6]. According to the instructions, classic I-AT should be started in normoventilated patients with a 10-min preoxygenation with 100% of O_2_ followed by blood collection to determine baseline arterial blood gas (ABG) [6]. Peripheral capillary O_2_ saturation and invasive blood pressure should be continuously monitored. After 10 min, another blood sample should be collected to determine ABG and the patient should be reconnected to the ventilator with pretest settings. The test is considered valid if there are no spontaneous respirations despite the rise of PaCO_2_ above 60 mmHg and over 20 mmHg above the baseline, and the patient is declared apneic. In case of desaturation or circulatory disturbances, an alternative AT method, such as hypoventilation with 100% O_2_, is recommended. To complete hypoventilation AT (H-AT) after preoxygenation, ventilator settings should be modified as follows: tidal volume and respiratory rate should be reduced to the minimal values at which the ventilator would not start backup apnea ventilation. These settings might differ depending on the type of ventilator. Positive endexpiratory pressure (PEEP) value should remain unchanged during this procedure. ABG levels should be monitored until test validity values are reached. During that period, patient’s chest and ventilator monitor should be carefully observed to exclude any ventilatory efforts by the patient. Similar approach was previously described by Ahlawat et al. [7].

The second AT ends the diagnostic procedure, and afterward, according to Polish guidelines, a BD declaration protocol is signed by three doctors. One should be an anesthesiology and intensive care specialist, another a neurology or neurosurgery specialist, and the third with a specialty not defined by guidelines.

Classic I-AT may be potentially harmful, and several serious complications including severe hypoxemia, hemodynamic instability, pneumothorax, and even cardiac arrest have been reported [7–9]. In addition, according to unpublished information obtained from Polish transplant coordinators, I-ATs are often followed by the deterioration of pulmonary function. If this status is confirmed, it necessitates the recommendation of another AT option with less harmful impact on pulmonary function. All complications more likely occur in cases of serious lung damage when very fast desaturation is observed [10, 11]. This may be caused by dramatic change of gas exchange conditions during the I-AT.

Usually, the patient is ventilated with a PEEP value of 5 cm H_2_O and rarely higher than 12 cm H_2_O. Additional pressure generated during the ventilation cycle results in mean airway pressure up to of approximately 10–15 cm H_2_O, which may keep the alveoli open even in moderately damaged lungs.

At the beginning of I-AT, after disconnection of the ventilator and placement of the insufflation catheter in the intubation tube, airway pressure rapidly falls close to 0 cm H_2_O. This may result in a rapid collapse of some alveoli; the others may collapse shortly after oxygen extraction to the pulmonary capillary vessels. Rapidly created pulmonary shunt may cause severe hypoxemia and result in a promptly preterm abortion of a test, which has often been reported [12]. Recruitment maneuvers and continuous positive airway pressure (CPAP) applied with some type of CPAP/PEEP valve [13], ventilator in CPAP mode, or with anesthesia machine circuit 11, may prevent such complications. Conversely, high CPAP/PEEP and recruitment may be theoretically risky and cause pneumothorax in some patients with pulmonary dysfunction, as suggested by van der Jagd et al. [14] in the commentary to the paper by Giani et al. [13].

In 2018, we published preliminary data from a multicenter trial based on 48 cases. In that study group, the ABG levels were mainly recorded at the beginning of AT and 10 min later at the end of AT, without an intermediate point at 5 min. The primary results indicated the possibility of pulmonary deterioration after completion of I-ATP and showed that CPAP-AT could be a safer option for the AT [15].

In the current study, the two AT methods were compared and the hypothesis regarding the negative impact of I-AT on pulmonary function was verified using a larger group of patients prior to the planned recommendation of a safer alternative, CPAP-AT, for the currently revised Polish BD criteria [16].

## Methods

The initial study plan for comparison of two AT methods was extended for the validation of CT angiography/perfusion in BD diagnosis and approved by the Bioethical Committee of Pomeranian Medical University. Family consent was not obtained because, except for children, it is not required according to Polish law. In this study, we retrospectively analyzed prospectively collected data from a basic group of 76 adult patients declared brain dead in three intensive care units from 2014 to 2017. In 60 patients, approximately 1.5 h after I-AT followed by BD diagnosis, a different method, CPAP-AT, was performed. Sixteen patients were excluded from the basic group for various reasons. In three cases, classic I-AT was aborted due to rapid desaturation defined as a drop in O_2_ saturation below 90%, after 1.5 min in the first case, 4 min in the second case, and 5 min in the third case. In addition, CPAP-AT was not attempted in eight patients because backup apnea ventilation could not be suspended when their ventilator was in the CPAP mode setting. In three cases, the documentation was incomplete, and in 2 cases the time span between I-AT and CPAP-AT was 12 h, which could have resulted in a change of pulmonary function.

PaO_2_ and PaCO_2_ were recorded prior to I-AT and CPAP-AT (time-point one, T1), 5 min after each test (time-point two, T2) and after 10 min prior to the end of tests (time-point three, T3). Changes in PaO_2_/FiO_2_ and PaCO_2_ during the first 5 min of each AT (Delta 1, difference of values measured at T2 minus T1) and the subsequent 5 min of AT (Delta 2, difference of values measured at T3 minus T2) were analyzed. In addition, systolic blood pressure and heart rate were continuously monitored. Minor changes in blood pressure were managed by modification of vasopressor infusion.

During I-AT, the ventilator was disconnected and oxygen was delivered at a flow of 6 L/min through the catheter inserted into the intubation tube. After BD was declared, approximately 1–1.5 h after the I-AT, a different method, CPAP-AT, was performed with a CPAP value of 10 cm H_2_O delivered with the ventilator (Evita XL, Draeger, Germany) after 10 min of preoxygenation. ABG samples were collected at T1, T2, and T3 at the beginning of each AT. Recruitment maneuvers were generally not performed due to the possibility of decrease in cerebral blood flow which we considered potentially dangerous for the patients before a final BD diagnosis [17]. All I-ATs and CPAP-ATs were performed by experienced anesthesiologists and intensive care specialists. Brain blood perfusion tests such as digital subtraction angiography transcranial Doppler (TCD), CT angiography and CT perfusion were performed in the majority of patients. These tests were not only part of the BD declaration procedure but were also used to validate CT angiography and CT perfusion for the diagnosis of BD.

The time elapsed between the onset of brainstem areflexia and formal declaration of BD was 35.7 ± 18.45 h (range 6–96 h). Ventilator settings prior to I-AT included FiO_2_ of 0.3–1.0 and PEEP of 3–15 cm H_2_O. In the majority of cases, PEEP was 5 cm H_2_O.

The patients in each group (I-AT and CPAP-AT) were further divided into two subgroups: non-hypoxemic (NH) with good lung function before the AT with PaO_2_/FiO_2_ index ≥ 200 mmHg, and hypoxemic (H) with poor lung function with PaO_2_/FiO_2_ index < 200 mmHg. The I-AT NH subgroup consisted of 50 patients, and CPAP-AT NH subgroup 43 patients. The I-AT H subgroup consisted of 10 patients, and CPAP-AT H subgroup 17 patients. PaO_2_ and PaCO_2_ at time-points T1, T2, and T3 were subsequently compared within these subgroups. Data were presented as mean ± standard deviation (SD) and standard error (SE). Groups at time-points Delta 1 and Delta 2 were compared using the Wilcoxon test for pairs of measurements at statistical significance level *p* < 0.05. Statistica 12.5PL software (StatSoft, Tulsa, OK, USA) was used for calculations and graph drawings.

## Results

I-AT and CPAP-AT were completed in 60 patients without serious complications such as severe desaturation, cardiac arrhythmia, or pneumothorax. Among the 60 patients included in this study, 63.3% were males and the mean age was 52.3 ± 13.26 years. The most common causes of death were cerebrovascular accidents (63%), traumatic brain injuries (25%), anoxic injuries (6%), and others (6%). Cessation of cerebral perfusion based on TCD or aortic arch angiography was confirmed in 38 cases, and electroencephalographic silence was observed in one case. In 21 patients, ancillary brain blood perfusion tests were not performed due to technical or organizational difficulties; however, this did not affect BD declaration.

The time elapsed between the onset of brainstem areflexia and formal declaration of BD was 35.7 ± 18.45 h (range 6–96 h). Ventilator settings prior to I-AT included FiO_2_ of 0.3–1.0 and PEEP of 3–15 cm H_2_O; in the majority of cases, PEEP was 5 cm H_2_O.

In addition to gasometrical tests, hemodynamic changes during the AT were analyzed. Hemodynamic parameters of the AT are shown in Table 1.

**Table 1:** Hemodynamic parameters during apnea test

Group	Heart rate (beats/min)	Systolic BP (mmHg)	Diastolic BP (mmHg)
I-AT NH	98 ± 18	142 ± 25	80 ± 14
I-AT H	100 ± 23	137 ± 19	78 ± 12
CPAP-AT NH	99 ± 22	138 ± 26	79 ± 15
CPAP-AT H	107 ± 22	127 ± 17	72 ± 11

*BP* Blood pressure, *CPAP-AT H* Continuous positive airway pressure apnea test in hypoxemic subgroup, *CPAP-AT NH* Continuous positive airway pressure apnea test in non-hypoxemic subgroup, *I-AT H* Insufflation apnea test in hypoxemic subgroup, *I-AT NH* Insufflation apnea test in non-hypoxemic subgroup

**Fig. 1:**
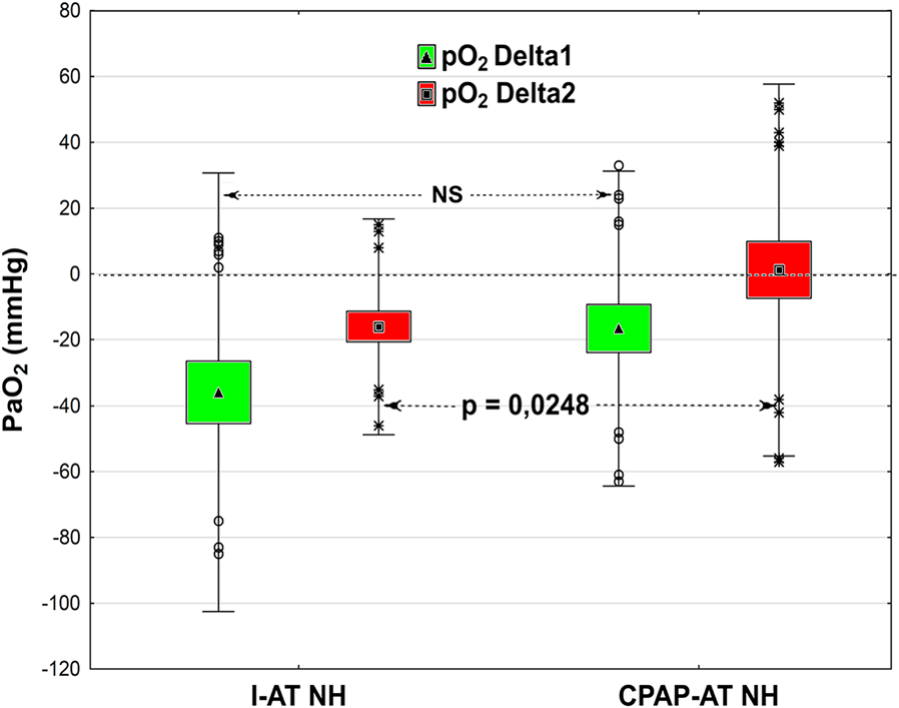
Change in PaO_2_/FiO_2_ in the NH subgroup during the first 5 min of the AT (Delta 1 = T2˗T1) and during subsequent 5 min (Delta 2 = T3˗T2). Box and whisker plots: data are represented as means (triangle or square in midpoint), SE (top and bottom of box) and SD (whiskers). Open circles or stars are outliers
  In single cases, increasing PaO_2_ was observed during the AT in both groups. The results of both ATs in the H subgroup are presented in Fig. 2. In the I-AT NH subgroup, a gradual decrease in PaO_2_/FiO_2_ was observed throughout AT but not in the CPAP-AT NH subgroup.

1. PaO_2_
  The results of both ATs in the NH subgroup are presented in Fig. 1.

**Fig. 2:**
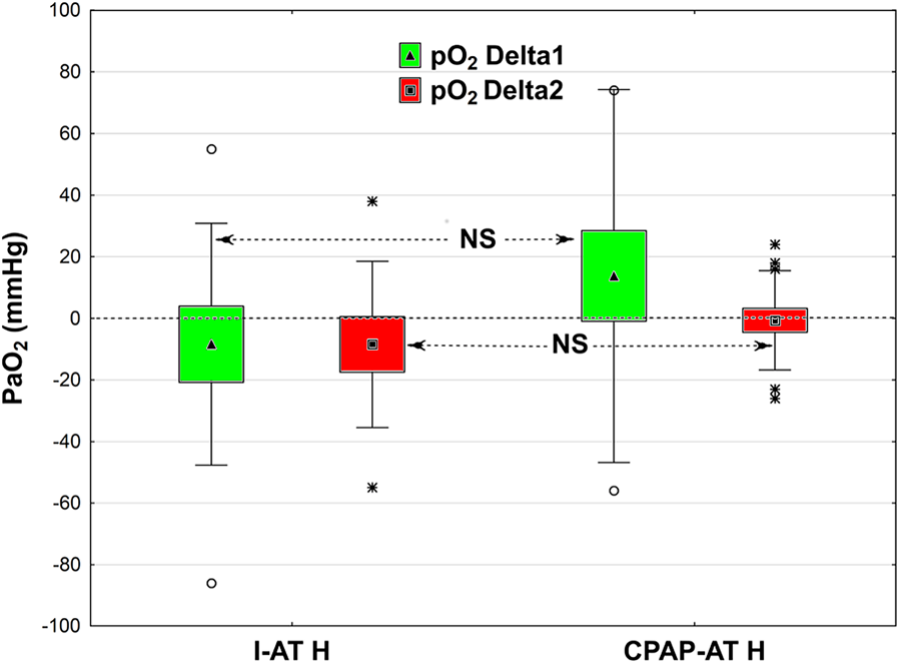
Change in PaO_2_/FiO_2_ during the first 5 min of AT (Delta 1 = T2˗T1) and during subsequent 5 min (Delta 2 = T3˗T2) in the two subgroups, I-AT H and CPAP-AT H. Box and whisker plots: data are represented as means (triangle or square in midpoint), SE (top and bottom of box) and SD (whiskers). Open circles or stars are outliers
2. pCO_2_
  The mean increase in CO_2_ in the NH subgroup was approximately 5 mmHg/min. The majority of patients in both subgroups after first 5 min of the test met the AT criteria; 50% slower increase in CO_2_ was observed during the next 5 min of the test. Results from the NH subgroups are presented in Fig. 3.

**Fig. 3:**
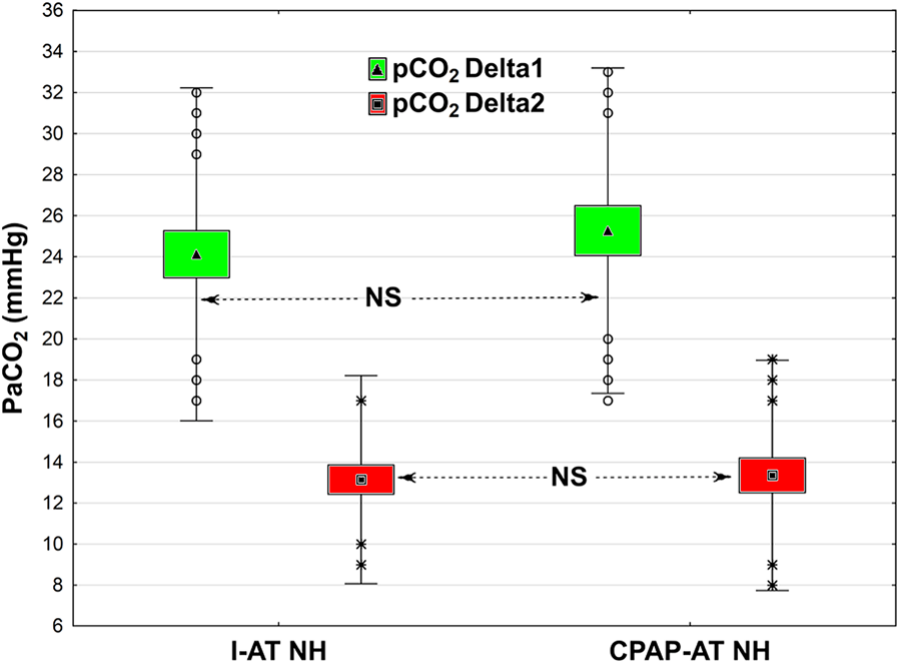
Change in PaCO_2_ during the first 5 min of AT (Delta 1 = T2˗T1) and during subsequent 5 min (Delta 2 = T3˗T2) in the two subgroups, I-AT NH and CPAP-AT NH. Box and whisker plots: data are represented as means (triangle or square in midpoint), SE (top and bottom of box) and SD (whiskers). Open circles or stars are outliers
  During the first 5 min, the mean increase in pCO_2_ in the H subgroups (mean increase over 5 mmHg CO_2_/min) was comparable to values in NH subgroups. Results from the H subgroups are presented in Fig. 4.

**Fig. 4:**
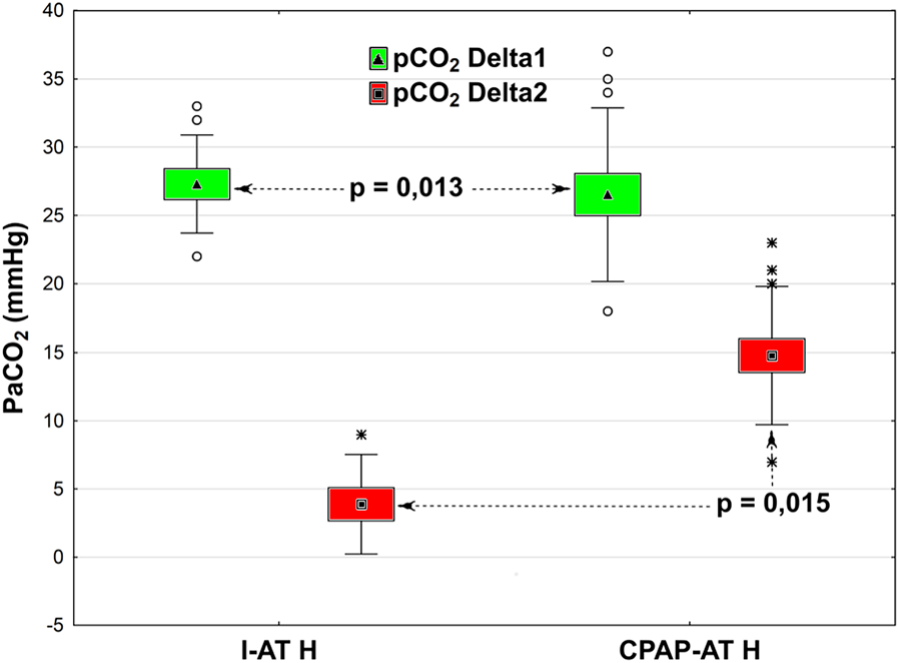
Change in PaCO_2_ during the first 5 min of AT (Delta 1 = T2˗T1) and during subsequent 5 min (Delta 2 = T3˗T2) in the two subgroups, I-AT H and CPAP-AT H. Box and whisker plots: data are represented as means (triangle or square in midpoint), SE (top and bottom of box) and SD (whiskers). Open circles or stars are outliers

In all three cases in which I-AT or CPAP-AT could not be completed due to rapid desaturation, the ATs with H-AT method were successfully performed without complications and BD diagnosis based purely on clinical criteria was possible. Of the 76 patients, 40 became organ donors. In 36 cases, organs were not obtained due to medical contraindications or family refusal.

## Discussion

The AT is the last clinical examination performed for the determination of death. It may be accompanied by severe desaturation and/or circulatory disturbances and therefore has to be aborted and modified, or in some cases, an additional instrumental confirmatory test has to be performed to complete the BD diagnosis [1]. Such complications were reported in 5% of cases [18], which is close to the results obtained in the present study, and I-AT had to be stopped in 3 of 76 cases. The rate of aborted ATs could potentially have been reduced if recruitment maneuvers were performed in patients with poor pulmonary function, but these were not included in our protocols, either before or after the AT. When this research was planned, the prevailing opinion based on literature was that greater damages than benefits could result from using recruitment maneuvers in patients with CNS pathology. During the research, our opinion changed and therefore we currently recommend recruitment maneuvers, especially in the I-AT option.

Probable collapse of a part of alveoli during I-AT may become permanent, especially if recruitment maneuvers are not subsequently performed. This may have explained translocation of some patients from the NH to H subgroup. Such phenomenon may not appear after I-AT if some type of ‘intrinsic PEEP’ was generated due to relatively big diameter of insufflation catheter or higher oxygen flow [19, 20].

In the I-AT NH subgroup, a gradual decrease in PaO_2_/FiO_2_ was observed throughout AT but not in the CPAP-AT NH subgroup. Similar findings were presented by Levesque et al. and Kramer et al. [21, 22]. Despite the lack of significant differences between PaO_2_/FiO_2_ values at the beginning and end of the tests in our study, this phenomenon could be particularly important in clinical terms in hypoxemic patients in whom PaO_2_/FiO_2_ is < 200 mmHg before the tests.

In the H subgroup, an increase in PaO_2_/FiO_2_ ≥ 200 mmHg was also observed in single cases during CPAP-AT, which requires further analysis. We cannot exclude the possibility of passive recruitment of borderline alveoli due to steady CPAP during the test. The CPAP value of 10 cm H_2_O could be close to, or sometimes higher than, mean airway pressure during ventilation and almost always higher than the PEEP value. Notably, the scatter of variables in the CPAP-AT H subgroup was considerable. This could be attributed to small sample size (17 cases) and differences in the severity of lung pathology. In patients with PaO_2_/FiO_2_ < 200 mmHg from the CPAP-AT group, aborting the test due to desaturation was not necessary. The use of this method may allow practitioners to avoid the necessity to use the ancillary tests in some cases during BD diagnosis. Conversely, this reduction of ancillary test number may not occur in Poland due to poor confidence of health care professionals and disagreement regarding BD diagnosis in some very active medical and religious authority groups. Polish physicians may feel more comfortable and safe if they could demonstrate the results of instrumental ancillary tests confirming BD diagnosis. Brain blood perfusion tests are considered the most evident for both sides of the discussion, the physicians and the relatives of the deceased. Obviously, regardless of the level of education, non-perfused tissue must be dead.

The rate of increase in CO_2_ was similar in both groups. During the first 5 min of the AT, the mean increase was approximately 5 mmHg/min, which allowed the test to be considered positive even at this stage. Similar findings were presented by Kramer et al. [22]. During the subsequent 5 min, a slower increase in CO_2_ was observed, but in the CPAP-AT H subgroup, this process was more dynamic (14.8 vs. 3.8 mmHg/5 min.). Thus, when the desired CO_2_ level was not achieved after 5 min in the CPAP-AT H subgroup, the increase in CO_2_/min was faster, which allowed for shorter AT.

Findings from the present study are currently used for the revision of the AT procedure in the Polish Brain Death Criteria. We recommend the following three versions of AT: CPAP-AT as a test of first choice, hypoventilation option as a second choice, and I-AT as a third choice. In addition, we recommend first ABG analysis after 5 min and then observe a definitive increase in PaCO_2_ value until the criteria of test validity are reached. This is consistent with the opinion of Kramer et al., based on the data obtained at the same time, but in organizationally different medical circumstances, that performance of ABGs at regular intervals shortens the AT duration and may avoid excessive pH reduction and subsequent hemodynamic effects [22]. We aborted the test in three cases due to hypoxemia resulting in failure rate similar to that reported by Lévesque and Kramer [21, 22]. However, we also noticed that H-AT may be a rescue option if AT cannot be completed with the other methods for any reason. This rarely mentioned option may circumvent the necessity to use ancillary tests which might be difficult to perform in unstable patients.

## Conclusions

The results from the present study show that I-AT may compromise pulmonary function in some cases, and this may be one of the reasons for the recommendation of a safer option, CPAP-AT, in the currently revised Polish BD criteria. The mean CO_2_ increase rate during AT was 5 mmHg/min, which in most patients would allow the test to be completed after just 5 min.

## Abbreviations

BD: Brain death
AT: Apnea test
I-AT: Insufflation apnea test
H-AT: Hypoventilation apnea test
NH: Non-hypoxemic
H: Hypoxemic
PaCO_2_: Arterial carbon dioxide tension
PEEP: Positive end-expiratory pressure
CPAP: Continuous positive airway pressure
CPAP-AT: Continuous positive airway pressure AT
ABG: Arterial blood gas
SpO_2_: Peripheral capillary O_2_ saturation
FiO_2_: Fractional inspired oxygen
PaO_2_/FiO_2_: Ratio of arterial oxygen tension (PaO_2_) to FiO_2_
SE: Standard error
SD: Standard deviation
